# Antibiotic regimen based on population analysis of residing persister cells eradicates *Staphylococcus epidermidis* biofilms

**DOI:** 10.1038/srep18578

**Published:** 2015-12-21

**Authors:** Shoufeng Yang, Iain D. Hay, David R. Cameron, Mary Speir, Bintao Cui, Feifei Su, Anton Y. Peleg, Trevor Lithgow, Margaret A. Deighton, Yue Qu

**Affiliations:** 1 Department of Infectious diseases, Wenzhou Central Hospital, Zhejiang, China 325000; 2 Dingli College of Clinical Medicine, Wenzhou Medical University, Zhejiang, China 325000; 3 Department of Microbiology, School of Medicine, Nursing and Health Sciences, Monash University, Clayton 3800, Victoria, Australia; 4 Department of Biochemistry and Molecular Biology, School of Medicine, Nursing and Health Sciences, Monash University, Clayton 3800, Victoria, Australia; 5 School of Applied Sciences, RMIT University, Plenty Road, Bundoora 3083, Victoria, Australia; 6 Department of Infectious Diseases, The Alfred Hospital, Melbourne 3004, Victoria, Australia

## Abstract

Biofilm formation is a major pathogenicity strategy of *Staphylococcus epidermidis* causing various medical-device infections. Persister cells have been implicated in treatment failure of such infections. We sought to profile bacterial subpopulations residing in *S. epidermidis* biofilms, and to establish persister-targeting treatment strategies to eradicate biofilms. Population analysis was performed by challenging single biofilm cells with antibiotics at increasing concentrations ranging from planktonic minimum bactericidal concentrations (MBCs) to biofilm MBCs (MBC_biofilm_). Two populations of “persister cells” were observed: bacteria that survived antibiotics at MBC_biofilm_ for 24/48 hours were referred to as dormant cells; those selected with antibiotics at 8 X MICs for 3 hours (excluding dormant cells) were defined as tolerant-but-killable (TBK) cells. Antibiotic regimens targeting dormant cells were tested *in vitro* for their efficacies in eradicating persister cells and intact biofilms. This study confirmed that there are at least three subpopulations within a *S. epidermidis* biofilm: normal cells, dormant cells, and TBK cells. Biofilms comprise more TBK cells and dormant cells than their log-planktonic counterparts. Using antibiotic regimens targeting dormant cells, *i.e*. effective antibiotics at MBC_biofilm_ for an extended period, might eradicate *S. epidermidis* biofilms. Potential uses for this strategy are in antibiotic lock techniques and inhaled aerosolized antibiotics.

Bacteria employ a variety of strategies to escape killing by antibiotics, including mutation, phenotypic variation, and change to a biofilm growth mode^1^,^2^,^3^. One form of phenotypic variation, known as persistence, is characterised by the presence of a subset of antibiotic-tolerant cells within a bacterial population. Persister cells pre-exist in most bacterial populations, including cultures at mid-log phase, stationary phase, and in biofilms^4^,^5^,^6^. The prevalence of persister cells in a population depends on its growth mode, the age of inocula, strain background, growth medium, and time course chosen for selection^6^,^7^,^8^. Persister cells display heterogeneity in growth rates and tolerance to various antibiotics^6^,^9^,^10^, though multidrug tolerance is not a consistent trait^11^. Transcriptome analysis suggests that persister cells have reduced expression of genes involved in metabolic pathways, biosynthesis pathways, and energy production^12^,^13^, which often leads to a dormancy status of cells. However, being entirely dormant is not necessarily a prerequisite for the formation of persister subpopulations^14^,^15^,^16^.

In clinical settings, bacteria grow predominantly as biofilms, following attachment and accumulation on biotic or abiotic surfaces; and they present group dynamics^17^,^18^,^19^. Bacteria within biofilms are highly tolerant to antibiotics, but the exact mechanisms behind this tolerance are complex and no single factor can fully account for this specific trait^20^,^21^,^22^. Persister cells are more prevalent in biofilms than in log-planktonic cultures and are thought to be responsible for the recalcitrance of many chronic infections, such as cystic fibrosis and chronic wound infections, to antibiotic treatment^1^,^3^,^9^,^23^. Although the role of persister cells in biofilm drug-tolerance has been reported by several studies^24^,^25^,^26^, quantitative evidence to support this is limited by the technical difficulty in excluding other biofilm-related factors when isolating persister cells. These include the presence of extracellular polymer substances (EPS), quorum-sensing (QS) factors and extracellular hydrolytic enzymes that may also protect the non-persister cells from the action of antibiotics^27^,^28^,^29^,^30^,^31^. Furthermore, the reported proportions of persister cells in bacterial biofilms varies, even when biofilms were cultured under similar *in vitro* conditions^32^. For instance, Shapiro *et al.* reported that the percentages of persister cells isolated from a *S. epidermidis* RP62A biofilm using levofloxacin or vancomycin were 28% and 94% respectively^33^. In contrast, a well-accepted relapsing biofilm infection model described by Lewis *et al.* proposed that biofilms comprised only ~0.1–1% persister cells^4^,^9^,^34^. This difference cannot be explained by stochasticity of the persister cell production in a bacterial population, but could be due to different bacterial strains used, or most likely different methodologies used to select persister cells^4^,^20^,^32^,^33^,^35^,^36^. Some researchers isolated biofilm persister cells by challenging the entire biofilm with antibiotics, and did not take into consideration the influence of those biofilm-related factors on antibiotic activity^33^,^37^,^38^,^39^. Others dissociated biofilm cells with sonication and/or vortexing, then challenged them with antibiotics to select persister cells^40^; this procedure however produced a relatively high proportion of bacterial clumps, which might present attributes resembling those of an intact biofilm. Many previous studies used antibiotics at 8–100 times minimum inhibitory concentration (MIC) and treatment periods between 2.5 hours (h) to 24 h to isolate persister cells^6^,^8^,^41^. Though these methods have been validated to select the generally defined “persister cells”, insufficient killing of all vulnerable cells in a bacterial population has been reported^33^,^42^,^43^. To obtain comparable data on the prevalence of persister cells within bacterial biofilms, an ideal method would require the separation of individual cells from other biofilm-related factors that affect the performance of antibiotics. Such a method should also specify concentrations of antibiotics and exposure times needed to effectively eradicate non-tolerant cells.

We have previously proposed the existence of three cell subpopulations residing within *S. epidermidis* biofilms; normal cells that are rapidly killed by antibiotics, tolerant-but-killable (TBK) cells that only respond to high concentrations of antibiotics, and dormant cells that resist very high concentrations of antibiotics; the latter two comprise persister cells^35^. In this study, we aimed to provide further support for our previous proposal, by examining the proportions of TBK and dormant cells in *S. epidermidis* biofilms. The specific aim was to accomplish two tasks in persister cell research recently raised by Lewis *et al.*^9^, but specifically related to *S. epidermidis* biofilms: 1) to detail the role of persister cells in antibiotic tolerance of biofilms; 2) to examine the possibility of eradication of persister cell and biofilms using conventional antibiotics *in vitro*.

## Results

### Validation of a method to isolate single cell populations from biofilms

**Figure 1:**
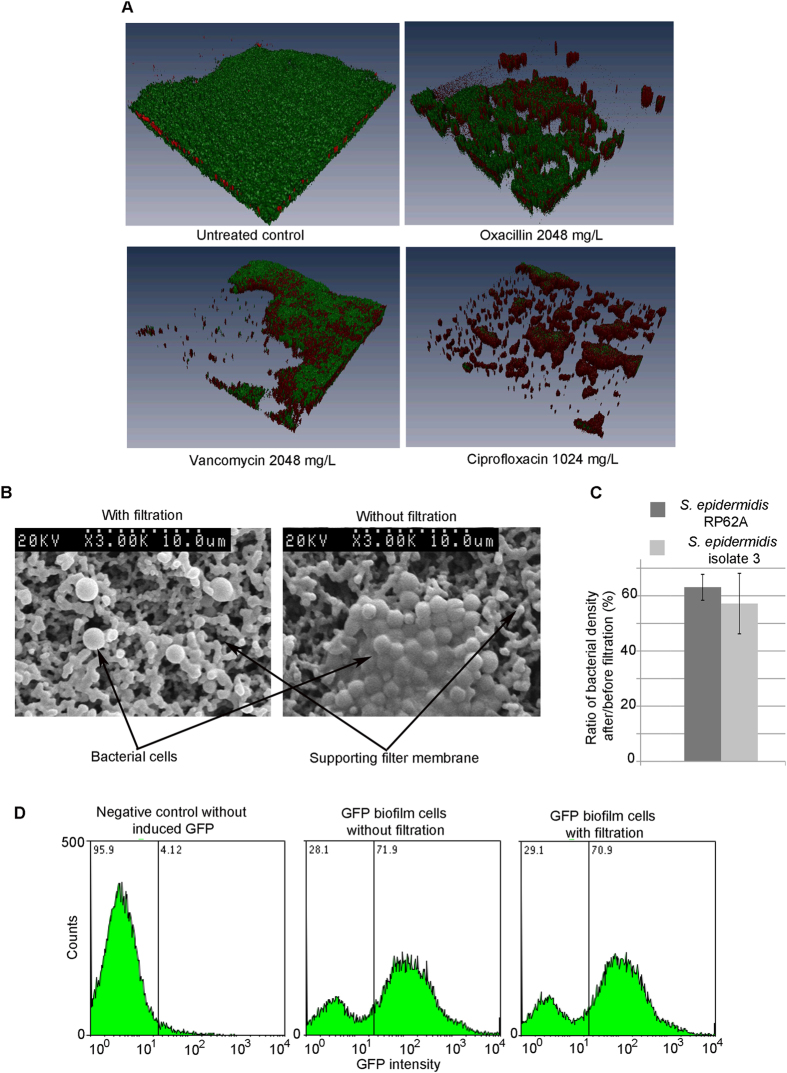
Validation of a new method to isolate single biofilm-embedded cells for persister cell studies. (**A**) CLSM of *S. epidermidis* RP62A biofilms treated with antibiotics at MBC_biofilm_ suggested isolation of single biofilm cells is necessary for persister cell quantification. (**B**) SEM of “single cells” isolated from mature biofilms with (left) or without filtration (right). (**C**) Quantitative comparison of densities (CFU/mL) of suspensions of single biofilm cells before and after filtration. (**D**) FACS of single biofilm cells before and after filtration.

Three-dimensional reconstruction of confocal laser scanning microscopy (CLSM; see supplementary information for experimental details) images showed a large number of survivors residing in biofilms after exposure to vancomycin and oxacillin at MBC_biofilm_ for 24 h (Fig. 1A); the percentage of survivor cells is inconsistent with that of persister cells in biofilms proposed by other researchers and ourselves^3^,^34^,^35^. The modified broth recovery-based biofilm MBC defined in our previous study was referred to as MBC_biofilm_ in this study^35^ (Table 1). This concentration of antibiotics was expected to eradicate normal cells and also “slow-replicating” cells residing in staphylococcal biofilms, leaving only very few viable persister cells^35^. We reasoned that dissociating biofilms and recovering single-cell population would provide a better measure of the proportion of persister cells than using intact biofilms. After mechanical disruption of *S. epidermidis* RP62A biofilms by scraping, pipetting, sonication, and vortexing, and passing the acquired cell suspensions through a 1.2 μm syringe filter, mostly single cells and diplococci were observed by SEM (Fig. 1B). In contrast, bacterial clumps consisting of more than 10 cells were frequently observed when the filtration step was not included (Fig. 1B). Viable counts performed on the bacterial suspensions obtained with or without filtration showed that approximately 45–65% of bacteria in the original suspension passed through the filter (Fig. 1C). GFP-expressing *S. epidermidis* RP62A cells were constructed and used in flow cytometry (see supplementary information) to measure levels of active and dormant subpopulations of biofilm-embedded cells. Biofilm cell suspensions collected with or without filtration showed similar percentage of GFP + (active) cells (70.9% versus 71.9%), suggesting that these ~45–65% cells are representative of the total population of embedded cells in the biofilm.

### Population analysis of *S. epidermidis* biofilm cells

**Figure 2:**
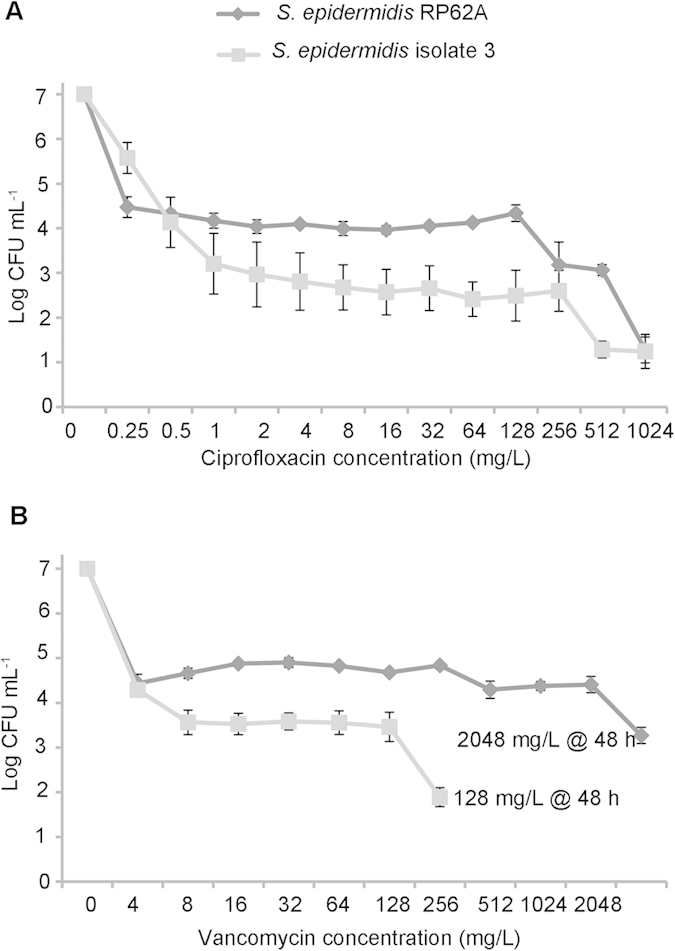
Population analysis of single biofilm-embedded cells. Population analysis profiling of single *S. epidermidis* biofilm cells with antibiotics at increasing concentrations identified three fractions of cells of different antibiotic tolerance. The first fraction represents the sensitive subpopulation; the majority of the population were killed following exposure to antibiotics at concentrations close to MBCs (4–8 mg/L for vancomycin and 0.5–1 mg/L for ciprofloxacin). The second fraction represents the persister cell population, which includes both TBK cells and dormant cells. This is a small population remaining tolerant to antibiotics at concentrations ranging from MBCs to sub-MBC_biofilm_. The third fraction became visible on further increasing the antibiotic concentration (for ciprofloxacin) and treatment period (for vancomycin) to eradicateTBK cells and to select dormant cells.

In contrast to the conventional notion that two subpopulations, antibiotic-susceptible bacteria and persister cells comprise a biofilm^8^,^15^,^44^, *S*. *epidermidis* biofilm cells presented three subpopulations in response to ciprofloxacin challenge (Fig. 2A). Most cells (>99.99%) were susceptible to ciprofloxacin at concentrations of 0.25–1 mg/L. The remaining cells, namely persister cells (~0.01% for the laboratory reference strain *S. epidermidis* RP62A and ~0.002% for a *S. epidermidis* clinical strain isolate 3), were tolerant of ciprofloxacin at concentrations ranging from 1 to 128 mg/L; most of these persister cells (~96.9% of RP62A persister cells and ~96.3% for isolate 3) could be further eradicated by ciprofloxacin at concentrations of 256–1024 mg/L. The third fraction of cells, dormant cells (~3.1% of RP62A persister cells and ~3.7% of isolate 3 persister cells), survived ciprofloxacin at a concentration as high as 1024 mg/L. Typical two-subpopulation patterns were established when vancomycin was used in place of ciprofloxacin. Over 99.9% of biofilm cells were susceptible, responding to vancomycin at concentrations below 4 mg/L. Persister cells (~0.06% for RP62A and 0.03% for isolate 3) demonstrated tolerance to vancomycin at concentrations ranging from 4 to 2048 mg/L (for RP62A) or 128 mg/L (for isolate 3). However, the two-subpopulation pattern could be extended as a three-subpopulation pattern when the incubation period was continued for vancomycin at MBC_biofilm_ for another 24 h (Fig. 2B). Upon 48 h exposure, only very few dormant cells from the persister cell population, approximately 1.8% for RP62A and 4.6% for isolate 3, survived vancomycin challenge. A similar pattern was found when oxacillin was used to select biofilm persister cells (data not shown). Thus we chose 24 h exposure for ciprofloxacin, or 48 h for vancomycin or oxacillin, at a concentration of MBC_biofilm_ (Table 1) to select for biofilm dormant cells.

### A biofilm population contains more tolerant-but-killable cells and dormant cells than a log-planktonic culture

**Figure 3:**
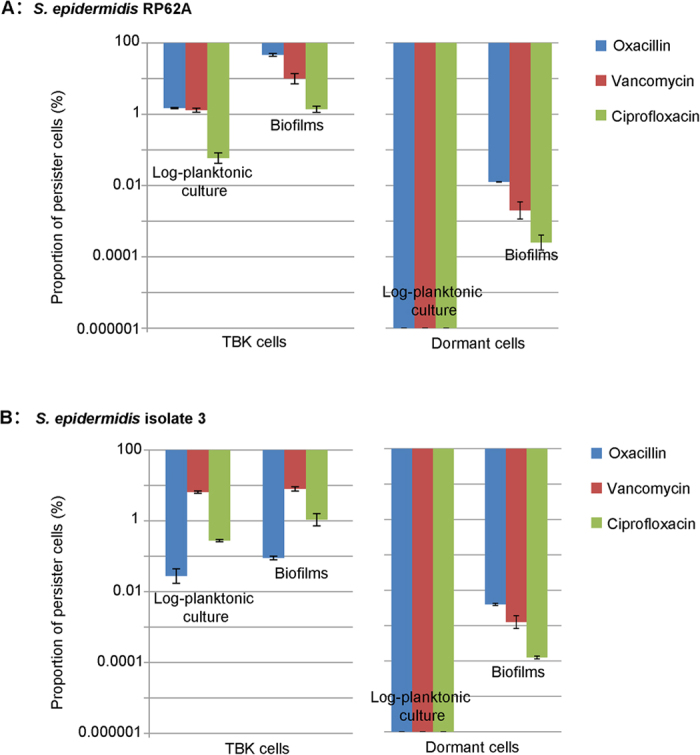
Proportions of *S. epidermidis* persister cells in two different growth modes. Total persister cells were isolated by exposing cell cultures to antibiotics at 8 X MIC for 3 h. Dormant cells were selected by exposing cell cultures to antibiotics at MBC_biofilm_ for 24 or 48 h. The proportion of TBK cells was calculated as the proportion of persister cells minus the proportion of dormant cells. Shown are the averages of three biological repeats in technical triplicates and the standard errors.

Dormant cells were not detected after exposure of log-planktonic cell cultures to any of the three antibiotics at the levels of MBC_biofilm_ for 24 or 48 h [limit of detection 10 colony forming units (CFU)/mL], however a substantial number of dormant cells (approximately 1 out of 10^6^ CFU to 1 out of 10^4^ CFU, corresponding to cell proportions 0.0001% to 0.01%) remained viable after exposure of single biofilm cells to these antibiotics (Fig. 3A,B, right panels). Fewer dormant cells were detected after exposure of RP62A or isolate 3 biofilm cells to ciprofloxacin than to vancomycin or oxacillin, with average cell proportions reached 0.00025% and 0.0001% for ciprofloxacin, 0.002% (*P* = 0.01) and 0.0013% (*P* = 0.004) for vancomycin, and 0.013% (*P* = 0.005) and 0.004% (*P* < 0.001) for oxacillin respectively (Fig. 3A,B).

TBK cells were isolated from both log-planktonic cultures and biofilms. Cell proportions of TBK cells ranged from 46.7% to 0.023% for individual isolates, different growth mode and antibiotics (Fig. 3A,B, left panels). For both *S. epidermidis* RP62A and isolate 3, biofilm cell populations generally produced a higher proportion of TBK cells than log-planktonic growth modes (*P* < 0.05) (Fig. 3A,B, left panels).

### Dormant cells are transiently resistant to antibiotics

It has been widely reported that generally defined “persister cells” are only transiently resistant to antibiotics. To determine whether the more stringently selected dormant cells were not genetically resistant to antibiotics, resuscitated dormant cells isolated from a *S. epidermidis* RP62A biofilm were examined for antibiotic susceptibilities using standard micro-dilution method. Regardless of the method used to isolate dormant cells, the MICs were not different from those of their parent cells (Table 2).

### SEM of TBK cells and dormant cells isolated from a *S. epidermidis* RP62A biofilm

**Figure 4:**
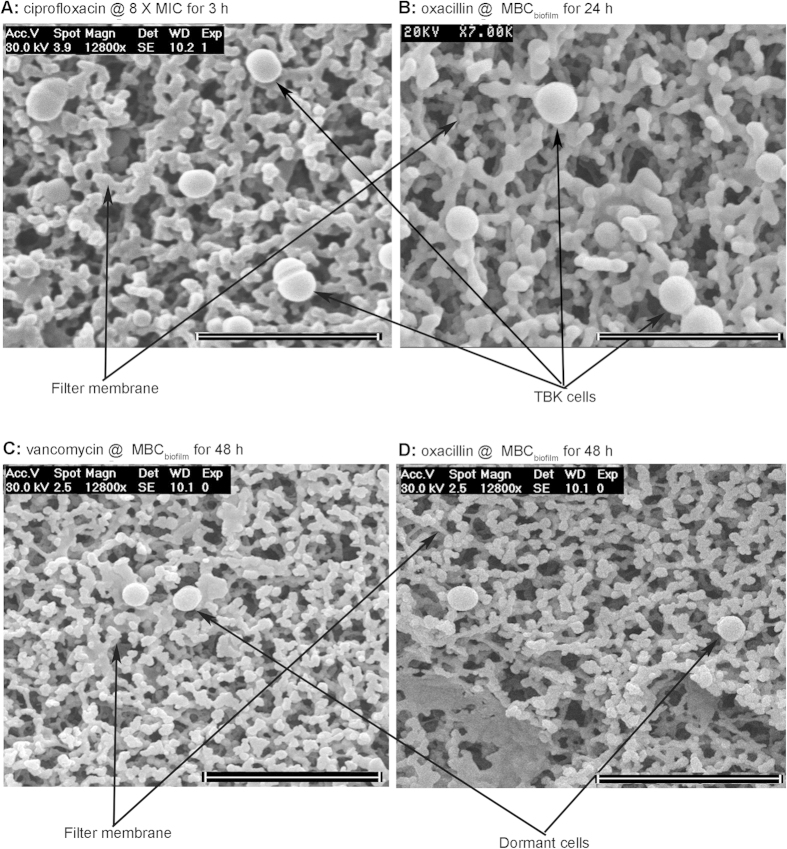
SEM of TBK cells and dormant cells isolated from *S. epidermidis* RP62A biofilms. (**A**) TBK cells isolated with ciprofloxacin (3 h, 8 x MIC). (**B**) TBK cells isolated with oxacillin (24 h, MBC_biofilm_). (**C**) Dormant cells isolated with vancomycin (48 h, MBC_biofilm_). (**D**) Dormant cells isolated with oxacillin (48 h, MBC_biofilm_). Scale bar = 5 μm.

Biofilm persister cells selected with either ciprofloxacin (3 h, 8 X MICs) or oxacillin (24 h, MBC_biofilm_) and comprising mostly TBK showed normal cell morphology when assessed by scanning electron microscopy (SEM, Fig. 4A,B). Some cells were in the process of cell division, indicating the presence of metabolically active dividing bacteria (Fig. 4A,B). In contrast, no dividing cells were found in the more stringently selected dormant cell population isolated using vancomycin and oxacillin at MBC_biofilm_ for 48 h. These cells appeared to have a smaller size, but with a normal shape (Fig. 4C,D).

### Antibiotic regimens targeting dormant cells are required to eradicate *S. epidermidis* biofilms

We hypothesized that choosing antibiotic regimens to target dormant cells might lead to eradication of *S. epidermidis* biofilms. These regimens can be achieved by using effective antibiotics at adequate concentrations for an extended period, as population analysis suggested the proportion of persister cell is related to the selecting antibiotic, its concentration and treatment period (Fig. 2). We examined the efficacy of ciprofloxacin and vancomycin, as single agents or in simultaneous combination for 48 h in completely killing persister cells and intact biofilms. At MBC_biofilm_, ciprofloxacin alone or combinations of ciprofloxacin and vancomycin, but not vancomycin alone, killed nearly all persister cells or biofilm cells of *S. epidermidis* isolate 3 (see signs in bold, Table 3). Survivors were found for *S. epidermidis* RP62A, which forms biofilms with more biomass than isolate 3 (data not shown). When 72 h and sequential combinations replaced 48 h and simultaneous combinations, ciprofloxacin or its combination with vancomycin at MBC_biofilm_ as the primary agent killed nearly all persister cells or biofilm-embedded cells of both isolates (in bold, Table 4). Sequential combinations with ciprofloxacin at MBC_biofilm_ as the primary agent for the first 24 h, followed by vancomycin at serum-achievable concentration (Table 1) for another 48 h on one occasion eradicated the persister cells and biofilms of isolate 3 (Table 4). In contrast, when antibiotics were used at serum-achievable concentrations, none of the treatments eradicated either persister cells or *S. epidermidis* biofilms, leaving a substantial number of survivor cells (Tables 3 and 4). To be noted, intact biofilms were occasionally more tolerant to antibiotic treatments than isolated persister cells. For instance, sequential combinations with ciprofloxacin (MBC_biofilm_) as the first agent followed by vancomycin (MBC_biofilm_) as the second agent killed almost all persister cells but left a substantial number of survivor cells within the biofilms.

## Discussion

Highlights of the current study include 1) development of a new method to recover single *S. epidermidis* biofilm-embedded cells free of biofilm-related antibiotic-compromising factors, 2) demonstrating the presence of three subpopulations of biofilm cells with regard to antibiotic susceptibility, and 3) suggesting strategies using antibiotic regimens to eradicate *S. epidermidis* biofilms.

To our knowledge, this is the first study that successfully isolated large numbers of single cells from bacterial biofilms to study their persister cell proportions. A combination of scraping, pipetting, sonication and vortexing was used to dissociate biofilm cells from the extracellular matrix, followed by a filtration step to select the single cells. The method was qualitatively and quantitatively validated by SEM, fluorescence-activated cell sorting (FACS), and viable counts before and after filtration. Antibiotic-compromising effects of some other biofilm-associated factors, such as extracellular hydrolytic enzymes and QS factors^27^,^28^,^29^,^30^,^31^, though not specifically examined, were believed to be minimized by resuspending the biofilm cell into fresh medium after removing from the substratum and before sonication, voxtexing and filtration.

Using the successfully isolated single biofilm cell population, this study provides quantitative and qualitative insights into the presence of antibiotic-tolerant cells within *S. epidermidis* biofilms. Population analysis suggested that biofilms contain TBK cell and dormant cell subpopulations in addition to normal cells. We showed that dormant cells constitute a very small subpopulation of cells that show little susceptibility to antibiotics, irrespective of antibiotic concentration. These cells were isolated under very stringent conditions that eradicated TBK cells. The rationality of using these stringent conditions is supported by our SEM findings. Both replicating and non-replicating persister cells were found when isolation was carried out following the method published by others^6^,^8^,^41^; however only non-dividing dormant cells were observed when specific concentrations and exposure times based on a pre-determined population analysis profile were introduced (Figs 2 and 4). TBK cells represent a much larger subpopulation than dormant cells. These cells have reduced antibiotic susceptibilities but remain eradicable with selected antibiotics. Viable but non-cultivable cells constitute the fourth cell population that might reside in biofilms^14^, however, this subpopulation is out of the scope of this study.

Our population profiling of biofilm persister cells based on antibiotic tolerance matches the persister classification by Babalan *et al.* and others^7^,^10^,^42^,^45^. Babalan *et al.* categorized persister cells as either type I or type II^10^. Type I persister cells are a fully dormant sub-population that can only be isolated from stationary-phase cultures; type II persister cells are a fraction of cells in a slow-growth state that can be obtained from any stages of growth, including log-planktonic cultures^10^,^45^. Dormant cells described in the present study are similar to type I persister cells. These cells were mainly isolated from the biofilm, a growth mode that has similar levels of resistance to antibiotics as stationary-phase cultures; SEM images of dormant cells showed no evidence of cell division. TBK cells might characteristically match the continuously generated type II persister cells. In our study, TBK cells could be isolated from both biofilms and log-planktonic cultures and SEM clearly showed dividing cells. Our finding that a single cell population isolated from *S. epidermidis* biofilms contained more persister cells than log-planktonic cultures is consistent with other published studies^3^,^4^,^39^, however this study goes into greater detail than previous studies. The observation presented in this study that dormant cells are only found in the biofilm growth mode suggests their important role in the tolerance of biofilms to high concentrations of antibiotics.

Zhang *et al.* has suggested a key principal for successful treatment of chronic infections: to reduce the persister cells quickly to a sufficiently low number with effective drugs so that reversion from persister cells to a normal bacterial population will not occur before the treatment is continued^46^,^47^. By introducing this principal into our study, we proposed a new strategy to treat *S. epidermidis* biofilm: killing TBK cells with effective antibiotics within a short period, followed immediately by a dormant-cell-targeted antibiotic regimen. Our population profiling suggested that high doses of ciprofloxacin but not vancomycin or oxacillin eradicate *S. epidermidis* TBK cells within 24 h. This is in accordance with the high efficacy of ciprofloxacin against staphylococcal biofilms^48^,^49^. We also observed that the critical concentration, MBC_biofilm_ must be reached to completely kill the TBK and to expose dormant cells. To further eradicate dormant cells, we propose extending the treatment period to a continuous 48 h or 72 h (Tables 3 and 4). In our *in vitro* study, eradication of dormant cells and intact biofilms were both achieved with ciprofloxacin at MBC_biofilm,_ or on some occasions, combinations with ciprofloxacin at MBC_biofilm_ as the major agent. Extended incubation might “wake up” the persister cells from dormancy and recover their susceptibility to antibiotics before they can establish a new population^50^,^51^. We however cannot exclude the possibility that the killing of dormant cells is simply a consequence of a longer exposure to antibiotics.

We initially proposed that combining vancomycin and ciprofloxacin would be more efficient in killing persister cells than ciprofloxacin alone, based on the staggered tolerance spectrum of persister cells to vancomycin and ciprofloxacin, and also on conclusions from other studies^8^. Surprisingly, combinations of vancomycin and ciprofloxacin at MBC_biofilm_ generally demonstrated a lower efficacy in eradicating persister cells or intact biofilms than ciprofloxacin alone. This might be explained by the finding by Dorr *et al.* that stress from antibiotics can stimulate the formation of persister cells^52^. To be noted, there is also a slight difference in antibiotic tolerance between single biofilm cell populations and intact biofilms (Tables 3 and 4), suggesting the possible involvement of other biofilm-related factors, such as the presence of EPS, QS factors, and extracellular hydrolytic enzymes.

In summary, *S. epidermidis* biofilm persister cells comprise TBK cells and dormant cells; both subpopulations play important roles in biofilm drug tolerance. Selection of a suitable antibiotic, at adequate concentration, and an extended exposure time, should be considered in the treatment of *S. epidermidis* biofilm-related infections. Although the concentrations of antibiotics and treatment period used in this *in vitro* study might not be suitable for intravenous administration, they could be applied for other applications such as antibiotic lock solutions, inhaled aerosolized antibiotics or wound dressings^53^,^54^.

## Materials and Methods

### Bacterial strains and antibiotics

Two biofilm-positive *S. epidermidis* strains: RP62A (ATCC 35984), resistant to oxacillin but sensitive to vancomycin; and isolate 3, sensitive to oxacillin and vancomycin, were used in this study. *S. epidermidis* isolate 3 is a clinical isolate, originally obtained from a blood culture from a baby being cared for in the Neonatal Intensive Care Unit, Royal Women’s Hospital, Melbourne^55^. First line antibiotics, oxacillin, vancomycin, and ciprofloxacin used in this study were purchased from Sigma (Sigma–Aldrich, Sydney, Australia).

### Biofilm assays and isolation of single biofilm cells

In order to isolate single cells from *S. epidermidis* biofilms, we combined a filtration step with mechanical disruption to separate biofilm cells from biofilm-associated factors that might impact on antibiotic efficacy. *Staphylococcus epidermidis* biofilms were grown in 6-well microplates with Tryptone Soya Broth (TSB, Oxoid, Hampshire, England) as described by Deighton *et al.*^56^. Single biofilm cells were isolated as follows: after preparing and washing biofilms, one milliliter of Muller-Hinton broth (MHB, Oxoid, Hampshire, England) was added to each well, and the wells were scraped thoroughly with a FALCON^®^ cell scraper (Corning, Mexico). The resulting suspensions were mixed well by pipetting several times and transferred into a sterile tube, followed by vigorous vortexing. This step was repeated three times and the final volume of each suspension was adjusted to 25 mL with MHB. The tube was sonicated for 10 min using a sonication bath (42 KHZ, BRANSON 1510) and then vortexed at the highest speed for 2 min (30″ × 4) to break up cell aggregates. The suspensions were firmly pressed through 1.2 μm Acrodisc syringe filters. Muller-Hinton broth was chosen as it is recommended by Clinical Laboratory and Standard Institute (CLSI) for antibiotic susceptibility testing and has been used previously in persister cell studies^23^.

### Preparation of single planktonic cells at mid-log phase

Planktonic cultures at mid-log phase were prepared by inoculating one colony of overnight-grown *S. epidermidis* into 5 mL nutrient broth (NB, Oxoid, Hampshire, England). Cultures were grown at 37 °C for 4–5 h until the turbidity was equivalent to McFarland 0.5 standard (~1 × 10^8^ CFU/mL). Bacterial suspensions were then centrifuged at 4400 *g* for 5 minutes, washed twice with sterile phosphate buffered saline (PBS), re-suspended in MHB, followed by the same vortex and filtration procedures described for biofilm cells.

### Population analysis of biofilm embedded cells

A published method for population analysis profiling was modified for this study as persister cells are unable to pass their “resistant” trait to descendants under a stress-free condition and cannot re-grow into a new population such as a colony on agar plates containing antibiotics^51^,^57^. To profile the population of *S. epidermidis* RP62A and isolate 3 biofilm cells upon antibiotic exposure, suspensions of single cells at ~10^7^ CFU/mL were prepared with MHB containing vancomycin or ciprofloxacin at increasing concentrations ranging from planktonic MBCs to MBC_biofilm_ (see below). Viable counts were performed after exposure of biofilm cells to vancomycin and ciprofloxacin for 24 h respectively, or exposure to vancomycin at MBC_biofilm_ (2048 mg/L) for 48 h. The rationale of selecting such concentrations of antibiotics was to ensure the effective killing of antibiotic-sensitive cells and TBK cells in the bacterial populations. MBCs of antibiotics for planktonic cells at mid-log phase were determined by the broth micro-dilution method recommended by CLSI^58^. MBC_biofilm_ of oxacillin, vancomycin, and ciprofloxacin for biofilms formed by *S. epidermidis* RP62a and isolate 3 were determined in our previous study (Table 1)^35^. To avoid antibiotic carryover which is related to the usage of very high concentration of antibiotics that might hinder the recovery of viable cells on agar plates, bacterial suspensions were pelleted by centrifuging at 20,000 *g* for 5 minutes, washed three times with PBS before being plated on Typtone Soya Agar (TSA) plates. Colonies were counted after 72 h of incubation at 37 °C. The log_10_ of CFU/mL was plotted against the antibiotic concentration. The difference in the number of survivors after exposure of cells to two antibiotic concentrations represents the fraction of cells that remains sensitive to the higher concentration but tolerant to the lower concentration of antibiotics.

### Quantification of tolerant-but-killable cells and dormant cells

We also used a previously reported method to quantify the generally defined persister cells from planktonic cultures at mid-log phase and single biofilm cell populations^6^,^41^. To isolate the generally defined “persister cell” population, which might comprise both TBK cells and dormant cells, suspensions of single cells at densities of ~10^8^ CFU/mL from planktonic cultures at mid-log phase and biofilms were exposed to antibiotics at 8 × MIC for 3 h^6^,^41^. This method has been shown to eradicate the majority of bacteria with normal susceptibilities^8^. Eight times of MIC of the selected antibiotics are slightly higher than their MBCs, but remain achievable in human serum (Table 1). Before and after the antibiotic challenge, 100 μL volumes of suspension were sampled, serially diluted with PBS and 10 μL was spotted on a TSA plate for cell counting. In some cases 100 μL volumes of the suspension were directly spread on agar plates to increase the sensitivity for detecting persister cells. Cultures were incubated for 72 h to maximize recovery of persister cells.

To isolate dormant cells, suspensions of single cells at mid-log phase or single biofilm cells were statically exposed to antibiotics at MBC_biofilm_ for the designated exposure time (37 °C, 24 h for ciprofloxacin and 48 h for vancomycin or oxacillin, based on our population analysis results). The same treatment periods were used for both log-planktonic cultures and biofilm cells to enable valid comparisons to be made. The numbers of survivors were determined by viable count as described above. The proportion of persister cells and dormant cells in different bacterial populations was calculated as follows: (bacterial density after antibiotic treatment)/(bacterial density before antibiotic treatment). The proportion of TBK cells was calculated as the proportion of persister cells minus the proportion of dormant cells.

### Antibiotic susceptibilities of dormant cells

In order to determine whether there was a resistance determinant passed from parent cells to the strictly selected dormant cells, antibiotic susceptibility tests were performed for descendants of dormant cells. Three to five colonies from the dormant cell quantification procedure (grown on TSA plate after antibiotic treatment) were inoculated directly into MHB and grown for 5 h. The resulting suspensions were used for the MIC tests, following the CLSI guideline.

### Scanning electron microscopy

Scanning electron microscopy was employed to examine the presence/absence of bacterial cell aggregations before and after filtration when isolating single cells from biofilms, and also to examine the morphology of biofilm persister cells and dormant cells. After isolating persister cells with ciprofloxacin (8 X MIC for 3 h), oxacillin (8 X MIC for 24 h), and dormant cells with oxacillin (MBC_biofilm_ for 48 h) and vancomycin (MBC_biofilm_ for 48 h) respectively, 100 μL of the suspensions were placed on filter membranes (0.2 μm or 0.45 μm) and air-dried overnight. The samples were sputter coated with 200 angstroms of gold using a Magnetron sputter coater (Dynavac, SC100M) and the trapped cells were viewed and imaged with scanning electron microscopes (Philips XL30 or Hitachi S570).

### Eradication of persister cells or intact biofilms with antibiotics

Intact *S. epidermidis* biofilms were formed in 96-well microplate^56^ and single biofilm cells were prepared as described above. Both biofilms and single biofilm cell populations were challenged with vancomycin and ciprofloxacin for 48 h, alone or in simultaneous combinations at MBC_biofilm_ or serum achievable concentration (see Table 3). In parallel, we challenged intact biofilms or isolated single biofilm cells with single agent for 72 h or two antibiotics sequentially for a total exposure period of 72 h. Two protocols were examined: vancomycin for 48 h and then ciprofloxacin for 24 h, or vice versa (Table 4). Viable counts were carried out for survivor persister cells as described earlier. To increase the detection sensitivity, a one mL volume of the treated suspension was pelleted, washed three times with PBS, and resuspended into 100 uL of PBS, followed by spreading and recovering on TSA plates. To perform viable count for survivors from intact biofilms, biofilms cells were scraped and removed from the bottom of a 96-well microplate and transferred in an Eppendorf tube. After dissociating the cell clumps by vortex and washing with PBS three times, the cells were plated on TSA plate and grown at 37 °C for three days.

## Additional Information

**How to cite this article**: Yang, S. *et al.* Antibiotic regimen based on population analysis of residing persister cells eradicates *Staphylococcus epidermidis* biofilms. *Sci. Rep.* **5**, 18578; doi: 10.1038/srep18578 (2015).

## Supplementary Material

Supplementary Information

## Figures and Tables

**Table 1:** Antibiotic susceptibilities of *S. epidermidis* and concentrations of antibiotics used to isolate antibiotic-tolerant cells (mg/L)*.

Abiotics	Oxacillin		Vancomycin		Ciprofloxacin	
Bacteria	RP62A	Isolate 3	RP62A	Isolate 3	RP62A	Isolate 3
MIC	4	0.12	2	2	0.12	0.12
MBC	8	0.25	2	2	0.12	0.12
Achievable concentration in serum)**	40		20–40		4.5	
8 x MIC (Persister-cell-isolating concentrations)	32	1	16	16	1	1
MBC_Biofilm_ (dormant- cell-isolating concentrations)	2048	2048	2048	128	1024	1024

*All MIC, MBC and MBC_Biofilm_ values are geometric means of the results of at least three independent measurements, taken to the closest doubling dilution.

**The highest serum achievable concentrations for antibiotics were as referred in the Manual of Clinical Microbiology 10^th^ edition.

**Table 2:** Antibiotic susceptibilities (MICs^a^) of *S epidermidis* RP62A resuscitated dormant cells (mg/L).

Bacterial origin	Oxacillin	Vancomycin	Ciprofloxacin
Parental cells	4	2	0.06
Dormant cells	4	2	0.06

^a^Each value is the geometric mean of three biological repeats in triplicates.

**Table 3:** Killing of intact biofilms or persister cells isolated from biofilms with single antibiotics or antibiotic combinations used simultaneously over a 48 h period.

*S. epidermidis*RP62A	VAN 8 mg/L	VAN 2048 mg/L	CIP 1 mg/L	CIP 1024 mg/L	VAN 8 mg/L + CIP 1 mg/L	VAN 8 mg/L + CIP 1024 mg/L	VAN2048 mg/L + CIP 1 mg/L	VAN 2048 mg/L + CIP 1024 mg/L
Persister cells	+++	++	++	+	++	+	+/++	+
Biofilms	+++	+++	+++	+	+++	++	+++	+/++
***S. epidermidis*** **Isolate 3**	**VAN 8 mg/L**	**VAN 128 mg/L**	**CIP 1 mg/L**	**CIP 1024 mg/L**	**VAN 8 mg/L + CIP 1 mg/L**	**VAN 8 mg/L + CIP 1024 mg/L**	**VAN 128 mg/L + CIP 1 mg/L**	**VAN 128 mg/L + CIP 1024 mg/L**
Persister cells	+++	++	++	**−**	+/++	−/±	+/++	**−/±**
Biofilms	+++	++	+++	**−**	+++	**−/±**	+/++	**−/±**

−: no survivor bacterium recovered from single biofilm cell populations (~1 × 10^7^ CFU) or intact biofilm populations (~1 × 10^8^ CFU).

±: 1 survivor cells recovered from single biofilm cell populations or from intact biofilm population.

+: 2–10 survivor cells recovered from single biofilm cell populations or 2–100 survivor cells recovered from intact biofilm population.

++: 11–100 survivor cells recovered from single biofilm cell populations or 101–1000 cells from intact biofilm population.

+++: >100 survivor cells recovered from single biofilm cell populations or >1000 cells from intact biofilm population.

**: experimental repeats showed variable results.

**Table 4:** Killing of intact biofilms or persister cells isolated from biofilms with single antibiotics or antibiotic combinations used sequentially over a 72 h period.

*S. epidermidis*RP62A	VAN 8 mg/L	VAN 2048 mg/L	CIP 1 mg/L	CIP 1024 mg/L	VAN 8 mg/L + CIP 1 mg/L	VAN 8 mg/L + CIP 1024 mg/L	VAN 2048 mg/L + CIP 1 mg/L	VAN 2048 mg/L + CIP 1024 mg/L
Persister cells	++/+++	+/++	+/++	**−/±**	++/+++	++	+/++	**−/±**
Biofilms	+++	+/++	+++	**−/+**	+++	++	++	**−/+**
					CIP 1 mg/L + VAN 8 mg/L	CIP 1024 mg/L + VAN 8 mg/L	CIP 1 mg/L + VAN2048 mg/L	CIP 1024 mg/L + VAN 2048 mg/L
Persister cells					++/+++	±/+	+	−/+
Biofilms					+++	++	++	++
***S. epidermidis*** **isolate 3**	**VAN 8 mg/L**	**VAN 128 mg/L**	**CIP 1 mg/L**	**CIP 1024 mg/L**	**VAN 8 mg/L + CIP 1 mg/L**	**VAN 8 mg/L + CIP 1024 mg/L**	**VAN 128 mg/L + CIP 1 mg/L**	**VAN 128 mg/L + CIP 1024 mg/L**
Persister cells	+/++	+	++	**−**	+/++	+/++	±/+	**−**
Biofilms	+++	+	+++	**−**	++/+++	++	+/++	**−**
					CIP 1 mg/L + VAN 8 mg/L	CIP 1024 mg/L + VAN 8 mg/L	CIP 1 mg/L + VAN 128 mg/L	CIP 1024 mg/L + VAN 128 mg/L
Persister cells					+/++	**−/±**	±/+	**−/+**
Biofilms					+++	**−/+**	+/++	+

−: no survivor bacterium recovered from single biofilm cell populations (~1 × 10^7^ CFU) or intact biofilm populations (~1 × 10^8^ CFU).

±: 1 survivor cells recovered from single biofilm cell populations or from intact biofilm population.

+: 2–10 survivor cells recovered from single biofilm cell populations or 2–100 survivor cells recovered from intact biofilm population.

++: 11–100 survivor cells recovered from single biofilm cell populations or 101–1000 cells from intact biofilm population.

+++: >100 survivor cells recovered from single biofilm cell populations or >1000 cells from intact biofilm population.

/: experimental repeats showed variable results.
