# Supplementary material for: Antibiotic regimen based on population analysis of residing persister cells eradicates Staphylococcus epidermidis biofilms
Source: Sci Rep. 2015 Dec 21;5:18578. doi: 10.1038/srep18578 (PMC4685274; doi:10.1038/srep18578)
Supplement: Supplementary Information [file srep18578-s1.docx]

**Antibiotic regimen based on population analysis of residing persister cells eradicates *Staphylococcus epidermidis* biofilms**

**Shoufeng Yang^1,2^, Iain D. Hay^3^, David R. Cameron^3^, Mary Speir^4^, Bintao Cui^5^, Feifei Su^1,2^, Anton Y. Peleg^3,6^, Trevor Lithgow^3^, Margaret A. Deighton^5*^, and**

**Yue Qu^3,6*^**

**Supplementary materials**

**Validation of a method to obtain single cells embedded in a *S. epidermidis* RP62A**

**biofilm**

To validate the use of 1.2 μm Acrodisc syringe filters for removing bacterial clumps and retaining adequate cells for persister cell study, 100 µL aliquots were removed from biofilm cell suspensions before and after filtration, FACS (using fluorescence-labeling cells, see below) and viable counts were performed and the suspensions were examined by scanning electron microscopy.

**Confocal laser scanning microscopy (CLSM)**

Sterile silicone disks were transferred to a 24 well microplate containing 1 mL of a bacterial suspension (~10^7^ CFU/mL) in each well, followed by 24 h incubation at 37 °C to allow biofilms to grow on the disks. The silicone disks were then rinsed three times with 0.9% saline to remove planktonic bacteria. The bacterial biofilms were treated with drug-free MHB, oxacillin at 2048 mg/L in MHB, vancomycin at 2048 mg/L in MHB, or ciprofloxacin at 1024 mg/L in MHB for 24 h. The biofilms were then washed three times with 0.9% NaCl and stained with a LIVE/DEAD BacLight viability kit, containing 3.35 µM SYTO-9 and 20 µM propidium iodide (PI), at 22 °C for 15 min in the dark. After being washed twice with 0.9 % saline, the structure of the biofilms was immediately examined with an inverted confocal laser scanning microscope (Leica SP5). Three-dimensional images of bacterial biofilms were assembled using the software Amira 5.0.

**Electroporation of *S. epidermidis* RP62A with the GFP expressing plasmid pALC2084**

The plasmid pALC2084 contains the *gfp* gene under the control of an inducible *xyl/tetO* promoter, it also contains the *tet* repressor *tetR* [^1^](#_ENREF_59). To overcome the restriction-modification system of *S. epidermidis* the plasmid was first transferred into a DNA methyltransferase mutant *E. coli* mutant (DH10B) ^2^. The plasmid was isolated from DH10B and concentrated to at least 1 μg/μL. Electrocompetent *S. epidermidis* RP62A were generated by first growing cells to an optical density (OD) 600nm of 0.4 in 500 mL TSB. Cells were cooled on ice for 30 minutes and harvested by centrifugation at 5,000 x*g*. The cell pellet was gently washed 3 times in 500, 250 and 100 mL of sterile MQ H_2_O with centrifugation at 5000 x*g* between each wash. Cells were then washed twice in 10 mL and 5 mL of 10% Glycerol. Finally cells were gently resuspended in 1 mL 10% glycerol, 0.5 M sucrose, frozen in liquid nitrogen and stored at -80 °C unless used immediately. Cells (75 μl) were mixed with ~3 μg of plasmid DNA transferred to a 1 mm gap electroporation cuvette incubated at room temperature for 5 min and pulsed at 2.1 kV/cm, 100 Ω. One mL of TSB at room temperature was added to the cuvette and the mixture transferred to a 15 mL tube, incubated at 30 °C for 2 h. then plated on TSB agar containing 10 mg/L chloramphenicol and incubated at 30 °C for 48 h. Transformants were confirmed by colony PCR and plasmid isolation followed by restriction digestion.

**Flow cytometric analysis**

Biofilms of *S. epidermidis* RP62A were grown in TSB for 20 h and then exposed to anhydrotetracycline (50 ng/mL in TSB) for 2 h. Anhydrotetracycline at such a low concentration had no effect on *S. epidermidis* RP62A biofilm biomass or CFUs, upon 2 h or 24 h exposure. Biofilm cells were collected as described above, except PBS containing sodium periodate (128 µg/mL) and trypsin (10 µg/mL) were used instead of PBS alone. Our preliminary experiments showed neither sodium periodate (128 µg/mL) or trypsin (10 µg/mL) affect the viability of *S. epidermidis* RP62A. This cocktail solution, however can dissociate *S. epidermidis* RP62A biofilm cell clusters into single cells, as shown in Fig. S1. Approximately 1 X 10^7^ cells were suspended in 1× PBS before GFP fluorescence was determined by flow cytometric analysis (BD FACSCalibur). Cells were gated by forward and side scatter, and channel F1 was used to detect GFP fluorescence. 10,000 events/sample were counted. Weasel software (Walter and Eliza Hall Institute of Medical Research) was used for the analysis.

### Statistical analysis

Antibiotic susceptibility tests and quantification of persister or dormant cells were carried out in triplicate on at least two separate occasions. Log transformation was applied to the obtained data for statistical analysis. One-way ANOVA was used to compare proportion of persister cells, TBK cells and dormant cells in different growth modes. A *P*-value of 0.05 was accepted as the significant level.

**Figure legend**

Fig. S1 Fluorescence microcopy of biofilm cell populations isolated by vortex and sonication with or without additional treatment of NalO4 and trypsin.


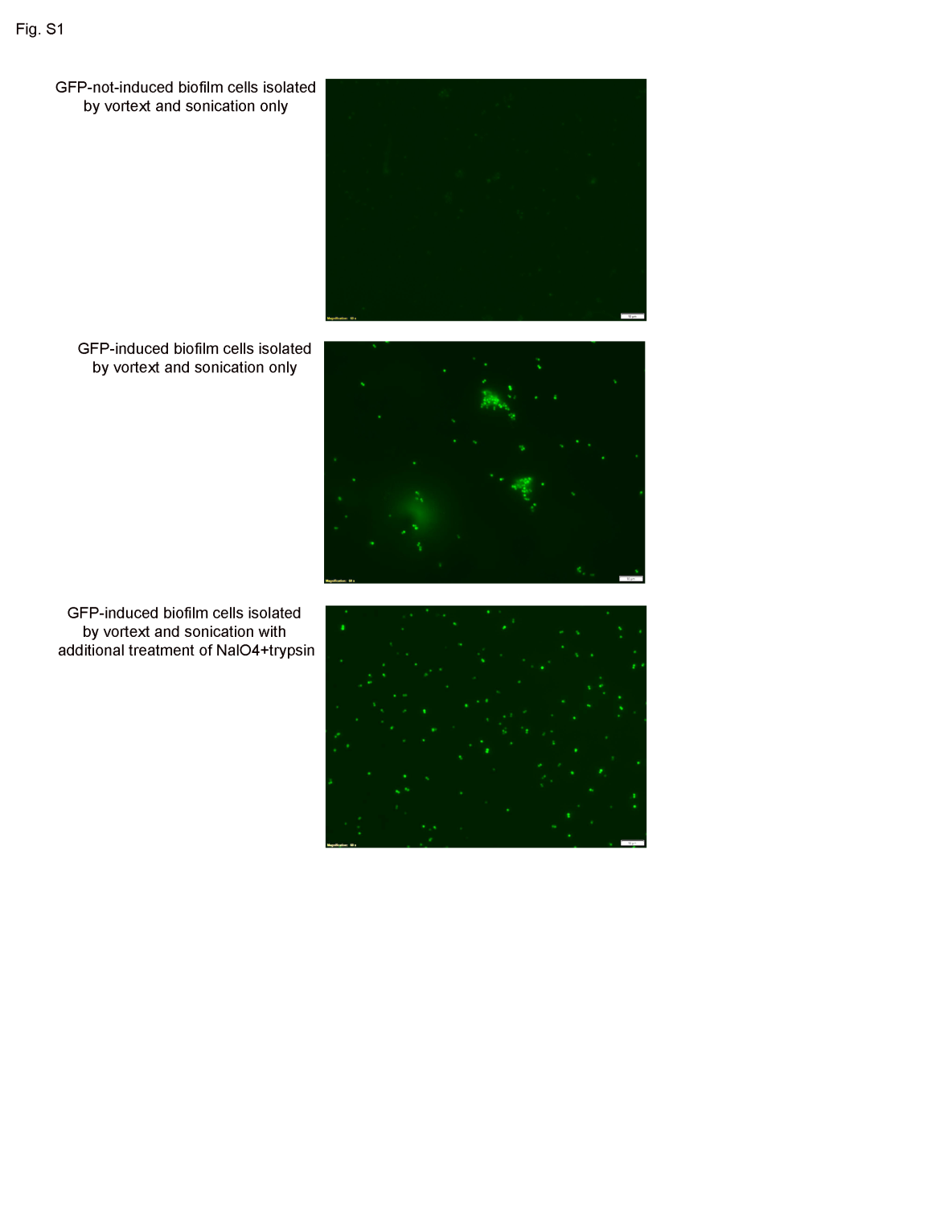


**References**

1. Bateman, B. T., Donegan, N. P., Jarry, T. M., Palma, M. & Cheung, A. L. Evaluation of a tetracycline-inducible promoter in *Staphylococcus aureus* in vitro and in vivo and its application in demonstrating the role of sigB in microcolony formation. *Infect. Immun.* **69**, 7851-7857 (2001).

1. Monk, I. R., Shah, I. M., Xu, M., Tan, M. W. & Foster, T. J. Transforming the untransformable: application of direct transformation to manipulate genetically *Staphylococcus aureus* and *Staphylococcus epidermidis*. *mBio* **3**, e00277-00211 (2012).
